# Food for Thought: Leptin and Hippocampal Synaptic Function

**DOI:** 10.3389/fphar.2022.882158

**Published:** 2022-06-17

**Authors:** Jenni Harvey

**Affiliations:** Division of Systems Medicine, Ninewells Hospital and Medical School, University of Dundee, Dundee, United Kingdom

**Keywords:** leptin, hippocampus (CA1), NMDA R, AMPA receptor, synaptic plasiticity

## Abstract

It is well documented that the endocrine hormone, leptin controls energy homeostasis by providing key signals to specific hypothalamic nuclei. However, our knowledge of leptin’s central actions has advanced considerably over the last 20 years, with the hippocampus now established as an important brain target for this hormone. Leptin receptors are highly localised to hippocampal synapses, and increasing evidence reveals that activation of synaptically located leptin receptors markedly impacts cognitive processes, and specifically hippocampal-dependent learning and memory. Here, we review the recent actions of leptin at hippocampal synapses and explore the consequences for brain health and disease.

## Introduction

A fundamental role of the adipocyte-derived hormone, leptin is to regulate energy balance by signalling the status of food stores to the hypothalamus. Leptin receptors (LepRs) are highly localised to specific hypothalamic nuclei, like the arcuate nucleus, that control energy homeostasis. When circulating leptin levels rise after eating, leptin binds to and activates arcuate nucleus LepRs which triggers a chain of events that culminates in feeling full (Friedman, 2019). However, the discovery that neuronal LepRs are not restricted to hypothalamic sites, fuelled speculation that the central functions of leptin were far more widespread. Studies using rodents with naturally occurring leptin or LepR gene mutations were pivotal in identifying a prime role for leptin in several extra-hypothalamic brain regions, including the hippocampus (Ahima et al., 1998; Ahima et al., 1999). Subsequent observations that leptin-insensitive rodents had significant impairments in hippocampal-dependent learning and memory processes suggested involvement of leptin in higher cognitive functions and raised the possibility that altering neuronal leptin responsiveness also influenced the functioning of hippocampal synapses (Li et al., 2002).

## Leptin and LepRs

Leptin is the product of the obese (ob) gene and the circulating leptin levels are directly proportional to body adiposity (Campfield et al., 1995). Adipocytes are the main source of leptin, but other peripheral tissues and central neurons can also generate this hormone (Schwartz et al., 1996; Ur et al., 2002). Leptin reaches the brain via transport across the blood brain barrier and evokes its biological actions by binding to leptin receptors (LepRs). Six different LepR isoforms (LepRa-f) exist. Although leptin binds to all isoforms, LepRb which is the long form, is the only isoform capable of activating the full spectrum of LepR-driven signalling pathways (Chen et al., 1996). In contrast, the shorter isoforms (LepRa,c,d,f) are implicated in the transport of leptin into the brain, whereas LepRe, which lacks a transmembrane domain, acts as a carrier for leptin in the plasma.

LepRs are class I cytokine receptors that signal via recruitment of janus tyrosine kinases (JAKs), and specifically JAK2 (Ihle, 1995). LepR-dependent activation of JAK2 leads to phosphorylation of tyrosine residues located within the LepR C-terminal. This sequence of events triggers activation of various downstream signalling pathways, including signal transducers and activators of transcription (STAT3), phosphoinositide 3-kinase (PI3K) and mitogen-activated protein kinase (MAPK), with hippocampal LepR capable of activating all of these downstream signalling pathways (Irving and Harvey, 2021).

## Hippocampal Expression of LepRs

In the CNS, specific hypothalamic neurons within the arcuate nucleus and ventromedial hypothalamus, express the highest density of LepRs, which is consistent with these nuclei being prime sites for controlling energy balance (Schwartz et al., 1996; Elmquist et al., 1998). However, LepR-positive immunoreactivity and LepR mRNA has been verified in other brain regions, like the hippocampal formation, which are not directly involved in control of energy homeostasis (Mercer et al., 1996; Elmquist et al., 1998; Hakansson et al., 1998). Interestingly, hippocampal LepR expression is regulated by metabolic hormones, like melatonin (Ni et al., 2015). Evidence also suggests that leptin may indirectly modulate hippocampal neuron energy homeostasis via regulation of mitochondrial function (Jin et al., 2018; Li et al., 2018).

Several studies have probed the cellular localisation of LepR in hippocampal neurons and found evidence for synaptic expression of LepRs (Shanley et al., 2002; O’Malley et al., 2007). Dual labelling immunocytochemistry detected a high degree of co-localisation between LepR and GluN2A-containing NMDA receptors (NMDARs), indicating that LepRs are localised to excitatory synapses (O’Malley et al., 2007). Together these findings point to a possible modulatory role for leptin at hippocampal excitatory synapses.

## Leptin Modulates Excitatory Synaptic Transmission at Hippocampal CA1 Synapses

NMDARs have minimal involvement in basal glutamatergic synaptic transmission. But during periods of high frequency stimulation, synaptic activation of NMDARs occurs, leading to persistent changes in synaptic efficacy and this process is known as long-term potentiation (LTP; Bliss and Collingridge, 1993). It is widely accepted that NMDAR activation is necessary for activity-dependent LTP at hippocampal Schaffer-Collateral (SC)-CA1 synapses (Collingridge et al., 1983). However, activation of NMDARs is also pivotal for other forms of hippocampal synaptic plasticity, such as long-term depression (LTD), which is induced by periods of low frequency stimulation. Activity-dependent changes in synaptic efficacy at hippocampal synapses, are thought to be key cellular processes underlying learning and memory (Bliss and Collingridge, 1993).

It is known that modifying NMDAR function is a key way of altering efficacy at hippocampal synapses. Significant evidence indicates that leptin regulates excitatory synaptic transmission at SC-CA1 synapses; effects that are highly dependent on NMDARs. At postnatal days 11–18 (P11-18), a transient depression of SC-CA1 synaptic transmission is evoked in leptin-treated brain slices (Shanley et al., 2001; Moult et al., 2010). Leptin also reduces synaptic transmission at SC-CA1 synapses earlier in development (P5-8), but this is a persistent process (leptin-induced LTD), as synaptic transmission remains depressed on leptin washout. The ability of leptin to reduce SC-CA1 synaptic transmission at P5-8 and P11-18 is prevented by NMDAR antagonists, indicating a central role for NMDARs in the depressant actions of leptin (Moult and Harvey, 2011).

At adult SC-CA1 synapses, leptin has completely opposite actions as a long-term increase in synaptic transmission (leptin-induced LTP) is observed in adult slices (Moult et al., 2010; Moult and Harvey, 2011). But NMDAR activation is also a pre-requisite for leptin’s effects in adult tissue, as leptin failed to influence synaptic transmission following antagonism of NMDARs. The effects of leptin were also absent when synaptic stimulation was paused, indicating involvement of synaptic NMDARs in leptin-induced LTP (Moult and Harvey, 2011). Earlier studies demonstrated that leptin potentiates NMDA-induced Ca2+ influx in hippocampal neurons and it facilitates pharmacologically isolated NMDA excitatory postsynaptic currents (EPSCN) in hippocampal slices (Shanley et al., 2001). Consequently, leptin is likely to drive enhancement of NMDAR function which in turn leads to long-lasting changes in excitatory synaptic strength.

Although NMDARs are required for the bi-directional effects of leptin at SC-CA1 synapses, detailed pharmacological analysis using subunit-specific NMDAR antagonists, uncovered that the molecular identity of NMDARs is pivotal for leptin’s divergent effects at different developmental stages. Thus, the synaptic depression induced by leptin at P5-8 or P11-18 requires selective activation of GluN2B-containing NMDARs. By contrast, GluN2A subunits underlie leptin-induced LTP in adult as this process was blocked by specific GluN2A, but not GluN2B antagonists (Moult and Harvey, 2011). This NMDAR subunit dependence parallels the reported developmental switch in synaptic NMDAR composition and the age-related decline in relative contribution of GluN2B subunits to synaptic NMDARs at SC-CA1 synapses (Monyer et al., 1994; Rumbaugh and Vicini, 1999; Moult and Harvey, 2011). Distinct LepR-dependent signalling pathways are also a factor in leptin’s bi-directional actions at SC-CA1 synapses, as PI3K mediates leptin-induced LTP in adult, whereas the leptin-driven reduction in synaptic transmission requires ERK-dependent signalling (Moult and Harvey, 2011).

Interestingly, LepR-driven enhancement of NMDAR function is implicated in leptin’s ability to restrict food intake. In the nucleus of the solitary tract (NTS), leptin enhances NMDA synaptic currents, which increases NTS neuron sensitivity to vagal stimulation and culminates in reduced food intake (Neyens et al., 2020). Facilitation of NMDA responses is also crucial for peripheral actions of this hormone, as leptin regulates pancreatic beta cell excitability by potentiating NMDA-dependent Ca2+ influx which triggers AMPK and trafficking of potassium channels to the membrane (Wu et al., 2017).

## Leptin Regulates Glutamate Receptor Trafficking Processes

Movement of AMPA receptors (AMPARs) into and out of synapses is key for cellular processes that modify excitatory synaptic strength, like LTP (Collingridge et al., 2004). However, NMDARs are also highly dynamic, with bi-directional changes in NMDAR movement linked to synaptic plasticity (Grosshans et al., 2002; Lau and Zuckin, 2007). Various neuromodulators, including leptin, can influence synaptic efficacy by modifying the mobility of glutamate receptors to and away from hippocampal synapses. Previous work uncovered that leptin facilitates NMDA-driven responses, as electrophysiological studies established that NMDAR synaptic currents are potentiated by leptin. Moreover, in primary hippocampal neurons treated with leptin, Ca2+ influx via NMDAR channels is amplified relative to control neurons (Shanley et al., 2001). However, studies in *Xenopus* oocytes revealed that leptin enhanced maximal NMDA-induced currents, in the absence of altered NMDAR channel kinetics, thereby signifying increased delivery of NMDARs to the membrane (Harvey et al., 2005). Leptin-driven NMDAR movement is implicated in the formation of new glutamatergic synapses, as hippocampal GluN2B surface expression is enhanced by leptin (Bland et al., 2020). In line with tyrosine phosphorylation mediating the anchoring of GluN2B subunits at synaptic sites (Vieira et al., 2020), tyrosine phosphorylation is also essential for leptin-driven movement of GluN2B subunits during synapse formation (Bland et al., 2020). Consequently, as facilitation of NMDA currents by leptin is blocked after tyrosine kinase inhibition (Harvey et al., 2005), phosphorylation of tyrosine residues may also be vital for NMDAR trafficking by leptin at later stages of postnatal development, although this remains to be explored.

**FIGURE 1:**
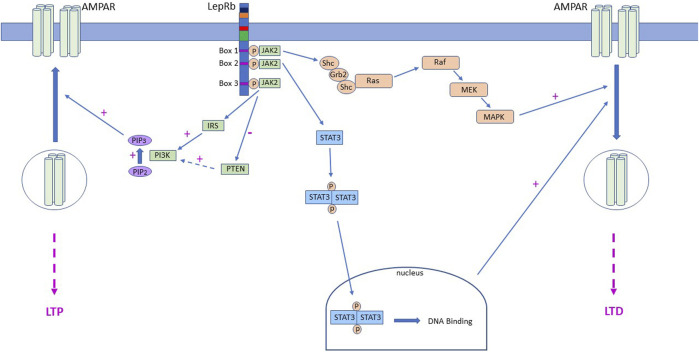
LepR activation drives alterations in AMPA receptor trafficking via different signalling mechanisms. Schematic representation of the signalling cascades activated downstream of LepRs that contribute to movement of AMPA receptors (AMPAR) to and from hippocampal CA1 synapses. In juvenile tissue, LepR stimulation of Ras-RAF-MAPK (ERK) signalling and subsequent removal of AMPAR from synapses, leads to induction of Ltd. at SC-CA1 synapses. Leptin is also capable of stimulating PI3K activity which increases intracellular PIP3 levels and drives delivery of GluA2-lacking AMPA receptors into TA-CA1 synapses, resulting in induction of LTP. Conversely, in adult hippocampus, LepRb activation drives inhibition of PTEN, which in turn elevates PIP3 levels and insertion of GluA2-lacking AMPARs into SC-CA1 synapses. In contrast, at adult TA-CA1 synapses, activation of LepRb stimulates canonical JAK2-STAT3 signalling which drives removal of synaptic AMPARs and subsequent LTD.

Persistent changes in hippocampal synaptic strength involve delivery or removal of synaptic AMPARs (Collingridge et al., 2004; Herring and Nicoll, 2016), with a short-lasting shift in synaptic AMPAR subunit composition reported in some cases (Plant et al., 2006; Morita et al., 2013). Likewise, leptin-induced LTP at adult SC-CA1 synapses is accompanied by delivery of AMPARs to synapses. Insertion of GluA2-lacking AMPARs is specifically involved as synaptic AMPAR rectification is raised after leptin treatment and pharmacological block of GluA2-lacking AMPARs prevents leptin-induced LTP (Moult et al., 2010). Additional evidence from studies in primary neurons and brain slices supports these findings as leptin boosts GluA1 levels at the plasma membrane and at hippocampal synapses (Moult et al., 2010). Mechanistically movement of AMPARs by leptin involves a rise in intracellular PIP3 levels which is attributed to inhibition of the phosphatase, PTEN by leptin (Figure 1). Interestingly an increase in PIP3 levels also underlies leptin-dependent synaptic insertion of AMPAR and subsequent induction of LTP at juvenile TA-CA1 synapses, however PI3K activation, not PTEN inhibition, drives this change in PIP3 levels (Luo et al., 2015).

## Regulation of Temporoammonic (TA)-CA1 Synapses by Leptin

Although the classical SC input to CA1 neurons is one of the most well-studied synaptic connections, direct innervation of CA1 pyramidal neurons via the TA input is also pivotal for hippocampal learning and memory (Vago et al., 2007). The TA pathway originates in entorhinal cortex layer III and extends to hippocampal stratum-moleculare where it forms synapses onto CA1 distal dendrites. Increasing evidence indicates that the two discrete inputs onto CA1 neurons have distinct functions, and that divergent mechanisms regulate SC-CA1 and TA-CA1 synapses. Thus, monoamines like dopamine depress excitatory TA-CA1 synapses but fail to affect SC-CA1 synapses (Otmakhova and Lisman 1999). Differential effects of other monoamines, including serotonin and noradrenaline, have been observed at the two CA1 synapses (Otmakhova et al., 2005). Electrophysiological studies indicate that leptin modifies TA-CA1 and SC-CA1 synaptic efficacy, but that it exhibits directly opposing actions at the two synapses. At juvenile TA-CA1 synapses leptin induces LTP, whereas a synaptic depression occurs at juvenile SC-CA1 synapses (Moult and Harvey, 2011; Luo et al., 2015). Interestingly, although GluN2B-containing NMDARs mediate the differential effects of leptin at these synapses (Moult and Harvey, 2011), distinct signalling pathways are involved as PI3K underlies leptin-induced TA-CA1 LTP, whereas ERK is implicated in the SC-CA1 synaptic depression induced by leptin. Similarly, opposing actions of leptin have been observed in adult hippocampus, despite GluN2A-containing NMDARs being required for leptin’s effects at both CA1 synapses. Thus, leptin induces NMDA-dependent LTD at adult TA-CA1 synapses *via* a process requiring JAK-STAT3 signalling (McGregor et al., 2018). By contrast, inhibition of PTEN underlies leptin-induced LTP at adult SC-CA1 synapses (Moult et al., 2010; Figure 1). Interestingly, parallels exist between leptin-induced TA-CA1 LTD and activity-dependent SC-CA1 LTD as activation of JAK2-STAT3 signalling is required for both forms of synaptic plasticity (Nicolas et al., 2012; McGregor et al., 2018), however gene transcriptional changes are necessary for leptin-induced LTD but not for NMDA-LTD at SC-CA1 synapses.

## Regulation of Inhibitory GABAergic Synapses by Leptin

Several studies have ascertained that leptin also modifies central inhibitory synaptic connections. In the hypothalamus, leptin reduces GABAA-mediated inhibitory synaptic transmission onto proopiomelanocortin (POMC) neurons (Cowley et al., 2001; Munzberg et al., 2007; Vong et al., 2011), whereas increased GABAergic inhibitory tone is observed in leptin deficient ob/ob mice (Pinto et al., 2004). In rat insular cortex, leptin regulates pyramidal neuron excitability by facilitating GABA release (Murayama et al., 2019). In the developing hippocampus leptin promotes development of functional inhibitory networks as it enhances GABAergic synaptogenesis (Sahin et al., 2021) and controls chloride homeostasis by modifying KCC2 activity (Dumon et al., 2020). At later postnatal stages (P13-19), leptin enhances GABAA-mediated synaptic transmission onto CA1 pyramidal neurons *via* a PI3K-dependent mechanism (Solovyova et al., 2009). The ability of leptin to potentiate GABAergic synaptic transmission in developing neurons also requires PI3K signalling (Guimond et al., 2014). However, the signalling pathways activated downstream of PI3K, and mediate leptin’s effects on GABAergic synaptic transmission during development and postnatally remain to be determined.

## Alterations in Hippocampal Synaptic Responsiveness to Leptin

### Dietary Changes

Accumulating evidence indicates that neuronal sensitivity to leptin is influenced by various factors. Diurnal changes in plasma leptin levels give rise to alterations in neuronal LepR expression, which in turn influences the magnitude of leptin responses (Lin and Huang, 1997; Baskin et al., 1998). Diets that are high in fats (so-called Western diets) can lead to an obese phenotype concomitant with development of resistance to insulin and leptin (Morrison et al., 2009). High fat diets (HFD) interfere with the neuronal actions of leptin, including its effects at hippocampal synapses. Thus, the ability of leptin to induce LTP at SC-CA1 synapses and to stimulate STAT3 signalling is absent in mice fed an HFD, compared to those on standard chow (Mainardi et al., 2017). Diet-induced obesity also triggers increased astrocytic expression of LepRs (Koga et al., 2014). As astrocytic LepRs help maintain glutamatergic synaptic transmission and synaptic plasticity at CA1 synapses (Naranjo et al., 2020), dietary driven changes in astrocytic LepR expression are likely to drive modifications in hippocampal synaptic efficacy.

### Age-Related Alterations in Leptin Sensitivity

The ageing process is also coupled to altered neuronal sensitivity to leptin. Age-related variations in leptin-driven signalling occur in hypothalamic neurons (Scarpace et al., 2001), which may underlie the reduced effects of leptin on food intake in aged rats (Shek and Scarpace, 2000). Reduced brain uptake of leptin also manifests with increasing age, which is likely to impact overall sensitivity to leptin (Fernandez-Galaz et al., 2001). These age-related shifts in leptin sensitivity parallel the changes in body composition and energy homeostasis that occur during ageing. Indeed, elevations in body weight and adiposity occur as humans get older, with this increase in adiposity accompanied by increased circulating leptin levels which enhances the likelihood of developing leptin resistance (Petervari et al., 2014; Cunnane et al., 2020). Obesity-related leptin resistance influences overall brain health, as increasing evidence supports a link between obesity and neurodegenerative disease (see *Leptin and Neurodegenerative Disorders* Section). Moreover, midlife obesity is a significant risk factor for development of type II diabetes (T2D), and the link between T2D and increased AD risk is well established.

The reported alterations in leptin responsiveness linked to ageing are not restricted to metabolic tissues, as hippocampal leptin function also declines with age (Moult and Harvey, 2011; McGregor et al., 2018). Thus, electrophysiological analyses revealed that the magnitude of leptin-induced SC-CA1 LTP diminishes with age (Moult and Harvey, 2011). Interestingly, the ability of leptin to induce TA-CA1 LTD is completely absent in aged hippocampus (McGregor et al., 2018). Although age-related alterations in leptin potency could explain the lack of leptin effect, this is unlikely as leptin failed to induce TA-CA1 LTD over a wide concentration range. Moreover, delivery of a low frequency stimulation paradigm also failed to induce LTD, suggesting that TA-CA1 synapses have been modified with age, rendering them insensitive to LTD inducing patterns of synaptic activity (McGregor et al., 2017). Further studies are needed to fully assess the cellular changes responsible for the alterations in TA-CA1 synaptic responsiveness to leptin with age.

## Leptin and Neurodegenerative Disorders

Growing epidemiological evidence supports the notion that life-style choices and particularly those adopted in mid-life, influence neurodegenerative disease risk. Indeed, an elevated risk of Alzheimer’s disease (AD) is connected to mid-life weight gain or obesity (Hassing et al., 2009; Xu, et al., 2011; Gustafson, et al., 2012). As feeding behaviour and thus body weight is controlled by leptin, this implies a prominent role for this hormone in overall AD risk. In support of this, abnormal leptin levels manifest in AD patients, whereas correlations between plasma leptin levels and disease risk have been described in prospective studies (Power et al., 2001; Lieb et al., 2009; Bonda et al., 2014). Abnormal leptin function occurs in various AD rodent models (Fewlass et al., 2004), indicating potential contribution of leptin dysfunction in both human and rodent forms of AD.

**FIGURE 2:**
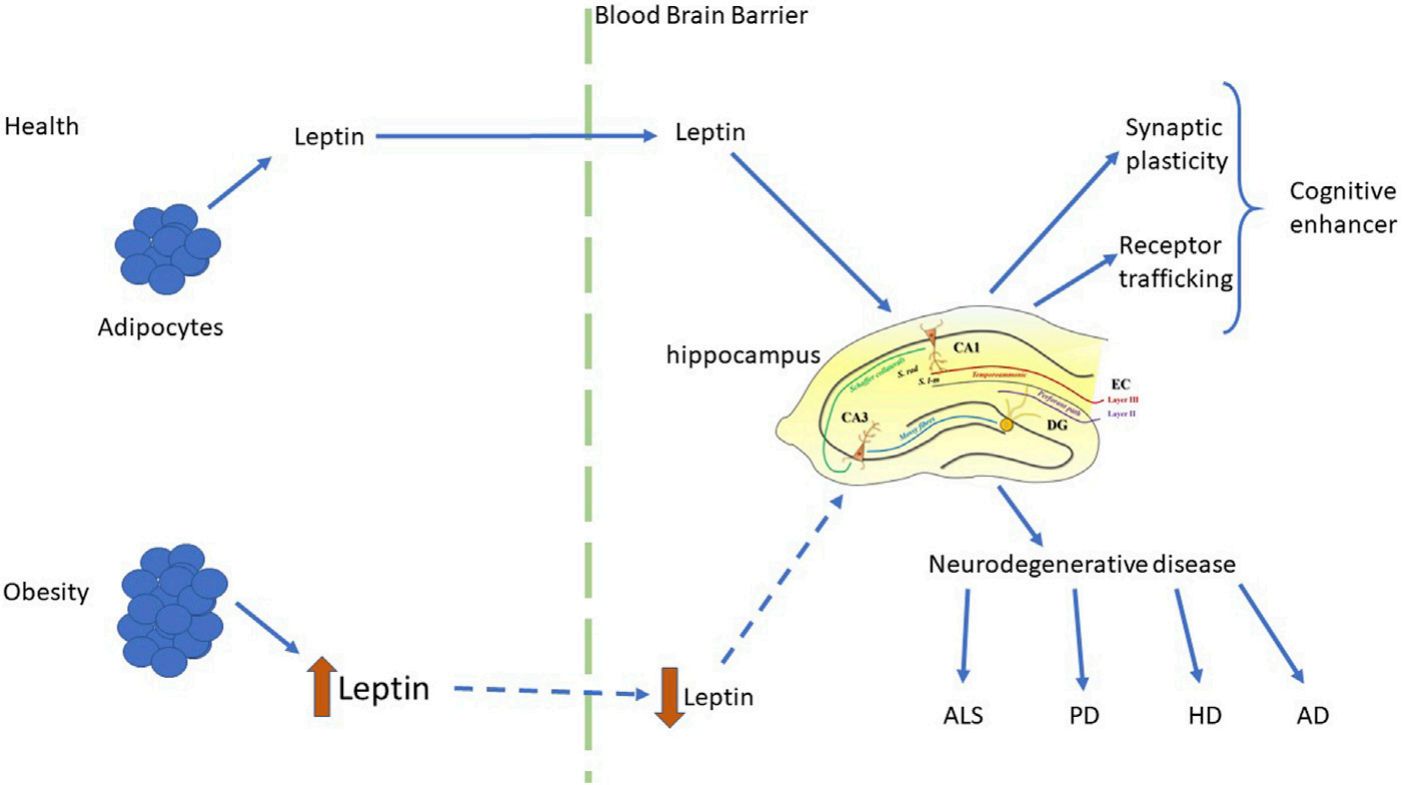
Regulation of hippocampal function by leptin in health and disease. Schematic representation of the key synaptic effects of leptin and the functional consequences of leptin-driven alterations in hippocampal function. In health, physiological levels of leptin are released from adipocytes and readily cross the blood brain barrier to reach the brain. Within the hippocampus, leptin has pro-cognitive actions via its ability to rapidly modulate hippocampal synaptic plasticity and glutamate receptor trafficking. In contrast, in the obese state leptin levels are elevated, leading to development of leptin resistance, and reduced transport of leptin into the brain. This subsequently leads to dysfunctions in the ability of leptin to regulate hippocampal synaptic function, which is associated with an increased risk of neurodegenerative disorders, such as AD, HD, ALS and PD.

Obesity is associated with an increased likelihood of other neurodegenerative disorders including Huntington’s disease (HD; Gaba et al., 2005; Procaccini et al., 2016), Parkinson’s disease (PD; Abbott et al., 2002), multiple sclerosis (Munger et al., 2009) and amyotrophic lateral sclerosis (ALS; Paganoni et al., 2011). A significant fall in circulating leptin levels has been reported in PD and HD patients (Evidente et al., 2001), suggesting correlation between leptin dysfunction and disease pathogenesis. Consequently, it is feasible that boosting leptin levels may have therapeutic benefit in these CNS-driven diseases. Although there is as yet no direct clinical evidence to support leptin’s efficacy in human neurodegenerative disorders, there is mounting evidence from pre-clinical studies that leptin has therapeutic potential as it has pro-cognitive and neuroprotective effects in cellular and rodent models of AD, PD and ALS (Greco et al., 2010; Doherty et al., 2013; Malekizadeh et al., 2017; Weng et al., 2007; Lim et al., 2014; Figure 2). It is also note-worthy that in addition to neurodegenerative disease, alterations in circulating leptin levels have been linked to other CNS disorders, such as childhood febrile seizures (Chen et al., 2020), suggesting that leptin’s ability to regulate hippocampal synaptic function has implications not only for brain health during the aging process, but also in childhood.

## Conclusion

There is now overwhelming evidence that the scope of leptin’s central actions extends beyond the hypothalamus, with hippocampal synapses a prime target for leptin’s regulatory actions. Leptin displays pro-cognitive actions as it modifies the efficacy of both SC-CA1 and TA-CA1 synaptic connections, which in turn impacts hippocampal-dependent memory. Leptin’s synaptic effects are highly age-dependent, with the polarity of leptin action driven by the activation of subunit-specific NMDARs and specific signalling molecules. Hippocampal CA1 synapse sensitivity to leptin also declines during the ageing process, which coincides with a fall in the functionality of metabolic hormonal systems. Age-related alterations in leptin function have implications for brain health, and specifically are correlated with an increased risk of neurodegenerative disease. Consequently, boosting brain levels of leptin may have therapeutic benefits in human neurodegenerative disorders, although this remains to be demonstrated clinically.
